# Defining and Treating Older Adults with Acute Myeloid Leukemia Who Are Ineligible for Intensive Therapies

**DOI:** 10.3389/fonc.2015.00280

**Published:** 2015-12-14

**Authors:** Kristen Pettit, Olatoyosi Odenike

**Affiliations:** ^1^Section of Hematology/Oncology, Department of Medicine, The University of Chicago, Chicago, IL, USA

**Keywords:** acute myeloid leukemia, elderly, fitness, older, treatment

## Abstract

Although acute myeloid leukemia (AML) is primarily a disease of older adults (age ≥60 years), the optimal treatment for older adults remains largely undefined. Intensive chemotherapy is rarely beneficial for frail older adults or those with poor-risk disease, but criteria that define fitness and/or appropriateness for intensive chemotherapy remain to be standardized. Evaluation of disease-related and patient-specific factors in the context of clinical decision making has therefore been largely subjective. A uniform approach to identify those patients most likely to benefit from intensive therapies is needed. Here, we review currently available objective measures to define older adults with AML who are ineligible for intensive chemotherapy, and discuss promising investigational approaches.

## Introduction

Acute myeloid leukemia (AML) is primarily a disease of older adults, with a median age at diagnosis of 66 years in the United States (1). Age has consistently emerged as an independent prognostic risk factor, with the prognosis declining particularly after 60 years of age (2–5). While the overall survival for younger patients with AML has improved somewhat over the past few decades, the prognosis for older patients remains consistently dismal (6, 7).

A variety of factors have been implicated in the poor outcomes of patients with advanced age. Older patients are more likely to have biologically poor-risk disease than their younger counterparts, including a higher incidence of poor-risk karyotypic abnormalities. Within each cytogenetic risk category, including intermediate and favorable risk groups, outcomes are worse with advancing age (8–11). In addition, older adults are more likely to develop AML in the setting of an antecedent hematologic disorder, which also confers a worse prognosis. Most cases of AML in patients over the age of 60, however, arise *de novo* and nearly half are cytogenetically normal (CN) (12, 13). In older patients with CN-AML, molecular variables can be helpful in refining risk (14–16).

Patient-specific factors also contribute to outcomes independent of AML characteristics. For example, worse performance status (10, 17, 18) and the presence of comorbid conditions have been associated with increased mortality and decreased response rates in this population (19, 20).

The tendency to manage older adults with less intensive measures may contribute to worse outcomes. Several studies have demonstrated improved survival for older patients receiving intensive induction chemotherapy compared to those receiving supportive care alone (2, 21). In the United States, however, <40% of older adults with AML receive chemotherapy for their disease (3). These data suggest a need for an improved understanding of factors that define ineligibility for an intensive treatment approach.

Defining this subset of patients who are not eligible for intensive therapy involves a great deal of subjectivity, and criteria have yet to be standardized across or within institutions. This review will focus on factors that should be taken into consideration to determine eligibility for an intensive treatment approach in AML and evolving treatment strategies, including investigational approaches, for older adults considered less fit for intensive induction therapy.

## Factors that Determine Eligibility for Intensive Induction Chemotherapy

### Physical Performance

Physical performance can be used to help predict outcomes in older patients with AML who are treated with induction chemotherapy. Methods available to quantitatively assess physical performance include the Eastern Cooperative Oncology Group performance status (ECOG PS), the Karnofsky performance status (KPS), and the short physical performance battery (SPPB). Retrospective analysis of data from clinical trials of patients treated with an intensive induction chemotherapy approach showed that in patients older than 65 years with poor ECOG PS of 2 or 3, outcomes declined drastically with age. For example, among patients with an ECOG PS of 3, the likelihood of early death increased from 0% in those <56 years to 29% in patients 56–65 years, and 82% in patients >75 years. However, for those with ECOG PS of 0–1, age appeared to have only a modest effect on the incidence of early death after induction chemotherapy (10). Another retrospective analysis of 998 patients age 65 years or older who underwent induction chemotherapy reported 8-week mortality rates of 23, 40, and 72% for patients with ECOG PS of 0–1, 2, and 3–4, respectively. The same groups had 1-year overall survival rates of 35, 25, and 7%, respectively (22). Similarly, the KPS has been shown to help predict outcomes in older patients (17, 23).

The SPPB (Table 1) is another objective measure of physical performance and has been shown to predict future disability, hospitalizations, and mortality among elderly patients in general, with or without a malignancy. The test is relatively simple to perform in the clinic in only a few minutes’ time and includes measures of balance, gait speed, and time to rise from a chair. Scores range from 0 through 12, with a score of 12 representing the most physically fit patient. A single-center study showed an association between lower SPPB score and increased risk of death specifically in patients older than 60 years with newly diagnosed AML undergoing intensive induction therapy. All evaluated patients had a reported EGOG PS of 0–1 at the time of evaluation. Those with SPPB scores <9 had a shorter median survival than those with scores >9 (6 versus 16.8 months, respectively). When analyzed as a continuous variable, each 2-point increase in SPPB score was associated with a 15% decrease in hazard ratio for death. This study showed that the SPPB is a valuable tool to further risk-stratify those with good ECOG PS who may have a lower functional reserve (20).

**Table 1:** **Short physical performance battery**.

Test	Instructions	Scoring
Chair stand test	Have patient cross their arms across their chest and stand from a seated position without the use of their arms five times, as quickly as they can. Measure the time that this takes the patient	<11.19 s = 4 11.20–13.69 s = 3 13.70–16.69 s = 2 >16.7 s = 1 Unable to complete = 0
Gait speed test	Measure the time required for the patient to walk 4 m at a normal pace (best out of two attempts)	<4.82 s = 4 4.82–6.20 s = 3 6.21–8.70 s = 2 >8.70 s = 1 Unable to complete = 0
**Balance tests**		
Side-by-side stand	Have patient stand with their feet together for 10 s	Able to complete = 1 Unable to complete = 0 (and do not proceed to semi-tandem or tandem stands)
Semi-tandem stand	Have patient stand with their feet staggered for 10 s	Able to complete = 1 Unable to complete = 0 (and do not proceed to tandem stand)
Tandem stand	Have patient stand with one foot directly in front of the other for as long as possible (up to 10 s)	10 s = 2 3–9 s = 1 <3 s = 0

### Comorbid Conditions

Comorbid conditions should also be taken into account when discussing AML management in older adults, as they portend worse prognoses and increased toxicity for patients undergoing intensive induction chemotherapy. Either the Charlson comorbidity index (CCI) or the hematopoietic cell transplantation-specific comorbidity index (HCT-CI) can be used to measure comorbid conditions quantitatively. Neither of these indices was initially designed for use in older patients with AML, but both have been studied in this population with varying results.

The CCI assigns point values for certain comorbid conditions, some of which are stratified for severity. The original CCI has been revised slightly for use in older adults with AML. A single-center retrospective study showed that patients with a CCI score >1 had a significantly lower chance of attaining a complete remission (CR) than those with a score of 0 or 1 (35 versus 63%). The group with higher scores also showed a trend toward higher 8-week mortality and lower 2-year survival (19, 20).

The HCT-CI (Table 2) was developed to improve the sensitivity of the CCI in the stem cell transplant setting, but has been evaluated as a tool to predict outcomes with intensive induction chemotherapy for AML as well. A retrospective study of 177 patients over the age of 60 years receiving induction chemotherapy for AML showed that HCT-CI scores of 0, 1–2, and >2 corresponded to early death rates of 3, 11, and 29%, respectively. The same groups had median overall survival times of 45, 31, and 19 weeks (24). A single-center study demonstrated that HCT-CI score ≥3 in older patients with AML was the single most significant predictor of overall survival and early death, even outweighing karyotype in that study (25).

**Table 2:** **HCT-CI (24)**.

Comorbidity	Definition	Score
Cardiac	Coronary artery disease, congestive heart failure with ejection fraction <50%	1
Arrhythmia	Atrial fibrillation, sick sinus syndrome, ventricular arrhythmias	1
Inflammatory bowel disease	Crohn’s disease or ulcerative colitis	1
Diabetes	Requiring treatment (either insulin or oral hypoglycemic)	1
Cerebrovascular accident	Cerebrovascular accident or transient ischemic attack	1
Psychiatric	Depression/anxiety requiring treatment (including psychotherapy)	1
Mild hepatic	Chronic hepatitis, bilirubin 1–1.5 × ULN, or AST/ALT 1–2.5 × ULN	1
Obesity	Body mass index >35 kg/m^2^	1
Infection	Documented infection or fever of unknown origin requiring antimicrobial treatment	1
Rheumatologic	Systemic lupus erythematosus, rheumatoid arthritis, polymyositis, mixed connective tissue disease, polymyalgia rheumatic	2
Peptic ulcer	Peptic ulcer disease requiring treatment	2
Moderate/severe renal	Serum creatinine > 2 mg/dL, on dialysis, or prior renal transplantation	2
Moderate pulmonary	DLCO and/or FEV_1_ > 65–80%, or dyspnea on slight activity	2
Prior solid tumor	Treated at any time in the past (excluding non-melanomatous skin cancer)	3
Heart valve	Any valvular disease (excluding mitral valve prolapse)	3
Severe pulmonary	DLCO and/or FEV_1_ < 65%, or dyspnea at rest, or requiring supplemental oxygen	3
Moderate/severe hepatic	Cirrhosis, bilirubin > 1.5 × ULN, or AST/ALT > 2.5 × ULN	3

*ALT, alanine transaminase; AST, aspartate transaminase; DLCO, diffusing capacity of the lung for carbon monoxide; FEV_1_, forced expiratory volume in 1 s; ULN, upper limit of normal*.

### Cognitive Function

Cognitive function should not be overlooked when considering treatment options for older adults with AML, as pretreatment cognitive impairment may increase the risk of complications during and after intensive therapy for AML (20). Data in this area are limited, but a few small studies have shown that cognitive impairment is common in this population and is an independent predictor of outcome. One study with a mean age of 70.8 years found that 31.5% of their patients had cognitive impairment at the time of diagnosis of AML (17). Another study from the same group showed that older patients with AML receiving induction chemotherapy with a modified mini-mental state exam score of <77 out of 100 had a median overall survival of 5.2 months compared to 15.6 months in those with a score ≥77 (20).

### Prognostic Models

Several prognostic models have been developed to risk-stratify and predict outcomes of patients undergoing induction chemotherapy based on patient and disease characteristics. An analysis of 2483 patients age 60 years or older enrolled in two UK trials showed that cytogenetic group, age, white blood cell count, performance status, and type of AML (*de novo* or secondary) were all associated with outcome in patients treated with either intensive or non-intensive regimens. When these factors were used to stratify patients into good, standard, and poor-risk groups, the 1-year survival rates were 53, 43, and 16%, respectively (26).

In a single-institution study of 998 patients age 65 years or older with AML treated with intensive chemotherapy, significant predictors of outcome were age ≥75 years, unfavorable cytogenetics, ECOG PS >2, antecedent hematologic disorder, lactate dehydrogenase (LDH) >600 IU/L, elevated creatinine, and treatment outside of a laminar air flow room. They went on to devise a scoring system based on the number of poor prognostic factors present. Those with none of the above risk factors had >60% CR rates, induction mortality of 10% within 8 weeks of treatment, and 1-year survival over 50%. This favorable group accounted for 20% of their study population. However, those with three or more risk factors had CR in <20%, induction mortality of >50%, and 1-year survival of <10%. This high-risk group accounted for 25–30% of their sample size (22).

Another prognostic model comes from a study of over 1400 older patients with AML who were otherwise healthy and were treated on a clinical trial with standard induction chemotherapy. This tool uses a formula including variables such as body temperature, hemoglobin, platelet count, LDH, age, type of AML (*de novo* or secondary), fibrinogen level, and molecular and cytogenetic features of the disease to predict probabilities for response and early mortality (27).

In a study of over 900 patients over the age of 60 years with AML who received standard induction chemotherapy followed by one cycle of consolidation, independent predictors of survival included karyotype, CD34 expression, white blood cell count at diagnosis, age, LDH, and nucleophosmin 1 (NPM-1) status. Karyotype was, by far, the most significant predictor of survival. Those with favorable risk cytogenetics fared the best, regardless of other factors, with 3-year overall survival rates of about 40%, while those with poor-risk cytogenetics had a dismal 3-year overall survival of only 3%. With this in mind, the authors devised a prognostic score to better define the risk for those in the intermediate cytogenetic category. Those with intermediate cytogenetics and more than three points on this scale were grouped into a category that they called “adverse intermediate,” while those with similar cytogenetics but three points or less were coined “good intermediate.” The 3-year survival rate for those in the adverse intermediate group was only 10.6%, as opposed to 30% for those in the good intermediate group (28).

At the moment, there is no consensus regarding a uniform set of guidelines that affirm fitness for intensive induction chemotherapy. The aforementioned prognostic scoring models, physical performance evaluation, comorbidity indices, and cognitive assessments can guide decision making in a more objective manner. However, validated guidelines are needed to standardize our treatment approaches globally. Several proposed guidelines have arisen from expert opinion and objective data, but no one algorithm has emerged as the standard in patient care. One such guideline developed by the Italian Society of Hematology (SIE), Italian Society of Experimental Hematology (SIES), and Italian Group for Bone Marrow Transplantation (GITMO) uses age, performance status, and comorbid burden to define fitness for intensive or non-intensive therapies (29). Table 3 demonstrates another evolving set of criteria for fitness, vulnerability, and frailty based on performance status, comorbidity assessment, and cognitive assessment that was recently proposed based on review of available evidence (30). Preliminary results of a separate consensus guideline, based on several patient-specific criteria and validated in a retrospective evaluation of 362 patients diagnosed and treated at multiple centers, were recently presented. This study demonstrated that the proposed criteria were able to predict for overall survival, regardless of the treatment modality. When combined with European LeukemiaNet (ELN) risk criteria (31), this model was able to identify a subgroup of fit, low/intermediate-I risk patients who did relatively well with a median overall survival of 20 months. Fit patients with intermediate-II risk or higher fared significantly worse, with a median overall survival of 8.5 months (32). This underscores the fact that these proposed tools still require the clinician to consider the patient’s fitness in the context of the disease biology. Some fit older patients with the highest risk disease may not derive sufficient benefit from standard induction chemotherapy to outweigh the risks, and these patients may be best served by consideration of alternative novel therapeutic strategies. A more uniform stratification of both fitness of the older patient for chemotherapy and appropriateness of that therapy in the context of disease biology would help inform clinical decision making as well as facilitate clinical trial design.

**Table 3:** **Evolving criteria for fitness in older adults with AML (30)**.

Risk category	Patient characteristics
Frail	ECOG PS ≥ 3
	Impaired activities of daily living
	Major comorbidity (CCI or HCT-CI > 1)
Vulnerable	ECOG PS < 3 with no major comorbidity
	Impaired objectively measured physical function (SPPB < 9)
	Impaired cognition (modified mini-mental state score <77)
Fit	Absence of all above risk factors

*CCI, Charlson comorbidity index; ECOG PS, Eastern Cooperative Oncology Group performance status; HCT-CI, hematopoietic cell transplantation-specific comorbidity index; SPPB, short physical performance battery*.

## Therapeutic Strategies for Patients Who are Unfit for Standard Induction Chemotherapy

Treatment options for patients deemed ineligible for intensive induction chemotherapy are few. Possible approaches may involve clinical trial participation, lower-intensity chemotherapeutics such as DNA hypomethylating agents, or supportive measures alone.

### DNA Hypomethylating Agents

The DNA hypomethylating agents decitabine and azacitidine are commonly used to treat this population. Both are approved by the US Food and Drug Administration (FDA) for the treatment of myelodysplastic syndrome (MDS). Decitabine is also approved by the European Medicines Agency (EMA) for AML, and azaciditine is approved for AML with 20–30% bone marrow blasts that arose from MDS. Both drugs are generally well tolerated and can provide some benefit in certain older patients with AML.

Decitabine has been investigated in the frontline setting in older adults with AML. A phase III, multicenter study was performed for patients >65 years with newly diagnosed AML comparing decitabine administered on a 5-day schedule in 28-day cycles to conventional care, which consisted of either best supportive care or low-dose cytarabine. The decitabine cohort demonstrated significantly higher CR rates (17.8 versus 7.8%). There was a trend toward increased median overall survival (7.7 versus 5 months, *p* = 0.11) that did not reach statistical significance. After another year of follow-up, the survival difference between the two groups did reach significance (*p* = 0.037) (33). The EMA approved decitabine for older adults with AML based on these data. However, the FDA declined a decision that has been criticized by some as representative of overly stringent statistical analysis (34). A phase II single-institution study of 53 older patients with AML not eligible for intensive therapy suggested that a higher CR rate can be obtained when decitabine is given for 10 consecutive days as opposed to 5-day MDS-like regimens. In fact, an impressive 47% of their patients attained a CR, and an additional 17% had no morphologic evidence of disease but had incomplete count recovery. Of those who achieved a CR, the median time to response was three cycles. One-year survival of even the poor-risk patients in this study was 30% (35). The results from this trial have led to the hypothesis that a 10-day schedule of administration may be more active for this agent in patients with AML and has led to further investigation of this schedule of decitabine in a recent cooperative group trial conducted in patients with AML >60 years of age (36).

Azacitidine has shown clinical activity in older patients with AML. In a phase III study (CALGB 9221) patients with MDS [45 had AML by current World Health Organization (WHO) criteria] were randomized to either azacitidine or best supportive care, with crossover to azacitidine permitted at the time of disease progression. The azacitidine arm demonstrated significantly improved response rates (60% overall response in the azacitidine group including 7% CR versus 0% in the supportive care arm). The response rates were similar between patients with MDS and AML in a subgroup analysis. The azacitidine group also reported significantly better quality of life measures, including fatigue, dyspnea, physical functioning, and psychosocial stress (37). In a landmark phase III study, AZA-001, patients were randomized to either azacitidine daily for 7 days of a 28-day cycle or a predefined, investigator’s choice conventional care regimen, which included best supportive care, low-dose cytarabine, or intensive induction chemotherapy. Most of the enrolled patients had MDS, but about one-third met WHO criteria for AML, with 20–30% blasts. A survival advantage was demonstrated for the azacitidine arm of the trial overall including the subgroup with WHO-defined AML. In that subgroup, median overall survival in the azacitidine arm was 24.5 versus 16 months in the conventional care regimen arm (38).

Preliminary results of the AZA-AML-001 study were recently presented. This phase III, multi-institution study compared azacitidine to conventional care regimens including intensive induction chemotherapy, low-dose cytarabine, or best supportive care in patients ≥65 years with AML and blast count >30%. The primary endpoint of median overall survival was 10.4 months in the azacitidine arm versus 6.5 months in the conventional care arm, which did not quite achieve statistical significance (*p* = 0.0829). There was a trend toward an improvement in the 1-year overall survival rates in the azacitidine arm as well (47 versus 34%) (39).

Emerging data suggest that certain subsets of patients may be more likely to respond to hypomethylating therapy. In an updated subgroup analysis of the AZA-AML-001 study, patients with morphologic dysplastic changes treated with azacitidine had twice the median overall survival than their morphologically similar counterparts treated with a conventional care regimen (12.7 versus 6.3 months, *p* = 0.0357) (40). There was some initial evidence that hypomethylating agents may be more effective in AML characterized by *DNMT3A* mutations; however, follow up studies were conflicting (41, 42). Recent reports also demonstrated that patients with *TET2* mutations are more sensitive to treatment with hypomethylating agents (43, 44). Further studies examining biological factors predicting response to epigenetic therapies are necessary and are ongoing.

### Low-Dose Cytarabine

Low-dose cytarabine represents another available option outside of a clinical trial for patients unfit for intensive therapy and remains a frequently used comparator or combination partner in clinical studies in this patient population (45). In a multicenter phase III trial, 217 patients with AML or high-risk MDS deemed unfit for intensive therapy were randomized to receive either low-dose cytarabine 20 mg twice daily for 10 days or hydroxyurea. The low-dose cytarabine group had a higher CR rate (18 versus 1%) and improved overall survival with an odds ratio of 0.60.

### Gemtuzumab Ozogamicin

In recent years, there has been a concerted effort to develop novel agents with better efficacy and toxicity profiles particularly for those patients who are considered unfit for standard induction approaches. Gemtuzumab ozogamicin (GO) is an antibody-drug conjugate that consists of an anti-CD33 antibody linked to calicheamicin. GO was granted accelerated FDA approval for patients with CD33^+^ AML in first relapse who were not candidates for cytotoxic chemotherapy based on several open-label studies showing improved outcomes in this population (46–48). However, the confirmatory SWOG 106 study, which involved the addition of GO to standard induction therapy in a separate population, namely untreated adults ≤60 years old, found an increase in 30-day mortality in this population which prompted the voluntary withdrawal of GO from the market, and thus it is no longer routinely available to older patients with AML (49).

Several subsequent studies focusing on older patients with AML have shown improved outcomes when GO is added to conventional therapy. In one randomized study comparing induction chemotherapy alone versus induction chemotherapy plus GO in older patients ranging from 51 to 84 years old with AML, there was no difference in response rates, early mortality, or toxicities between the two groups, but at 3-year follow up, there was a decreased relapse rate (68 versus 76%, *p* = 0.007) and improved survival (25 versus 20%, *p* = 0.05) in the group who received GO (50). Another trial randomized 495 older patients with AML ranging from 54 to 90 years old who were deemed inappropriate candidates for intensive therapy to low-dose cytarabine with or without GO. The addition of GO resulted in significantly improved remission rates (30 versus 17%, *p* = 0.006), but no improvement in mortality at 12 months (51).

The role of GO as post-remission therapy for older patients has also been investigated. A phase III multicenter study randomized patients over the age of 60 years in remission after intensive therapy to receive either three cycles of GO or no post-remission therapy. They found no difference in relapse rates, disease-free survival, or overall survival (52). The ultimate fate of this agent has yet to be determined, but many experts have vocally advocated for its reinstatement in our treatment armamentarium (53–55).

## Novel Agents Under Investigation

In recent years, there has been an increasing focus on molecularly targeted therapies in oncology, and AML is no exception. Several targeted small molecule inhibitors are under investigation for older patients with AML who are not fit for standard induction therapy. In general, these agents are hypothesized to be less toxic than traditional chemotherapy, and as such could be useful in specific molecular subsets of AML in less fit older adults either as single agents or in rationally designed combinations in the near future (Table 4).

**Table 4:** **Selected list of investigational agents**.

Agent	Mechanism of action	Phase of investigation	ClinicalTrials.gov identifier	Reference
**Drugs that target the microenvironment or leukemic stem cell**				
PF-04449913	Hedgehog inhibitor	I/II	NCT01546038	(56)
**Kinase inhibitors**				
Volasertib	PLK1 inhibitor	III	NCT01721876	(57, 58)
GSK2141795 + trametinib	RAS activation pathway inhibitor	II	NCT01907815	(59)
Quizartinib	FLT3 inhibitor	I/II	NCT01892371	(60, 61)
ASP2215	FLT3/AXL inhibitor	I	NCT02014558	(62)
Pevonedistat	NAE inhibitor	I	NCT01814826	(63, 64)
**Epigenetic therapies**				
Pracinostat	HDAC inhibitor	II	NCT01912274	(65)
Valproic acid	HDAC inhibitor	II	NCT00867672	(66, 67)
			NCT00414310	
Vosaroxin	Topoisomerase II inhibitor	I/II	NCT01893320	(68)
Bortezomib	NF-kB pathway inhibitor	II	NCT01420926	(36)
AG-120	IDH1	I	NCT02074839	(69)
AG-221	IDH2	I	NCT01915498	(70)
EPZ-5676	DOT1L	I	NCT02141828	(71)
SGI-110	DNA hypomethylator	I–II	NCT01261312	(72)
**Others**				
Selinexor	SINE	II	NCT02088541	(73)

*SINE, selective inhibitor of nuclear export*.

### Agents That Target the Microenvironment/Leukemia Stem Cell

PF-04449913 is an oral agent designed to inhibit the hedgehog signaling pathway, which has been shown to be aberrantly activated in AML cells. It is currently undergoing phase I/II trials in combination with induction chemotherapy in fit patients, and with either low-dose cytarabine or decitabine in unfit patients (56).

### Agents That Target Dysregulated Kinases or Signaling Pathways

Quizartinib, an FMS-like tyrosine kinase 3 (FLT3) receptor tyrosine kinase inhibitor, has shown promising results in patients with relapsed refractory AML, particularly those patients harboring an FLT3-internal tandem duplication (ITD). In a phase I study, responses [including CR, CR with incomplete platelet recovery (CRp), CR with incomplete hematologic recovery (CRi) (74), and partial remission (PR)] were seen in 30% of all patients and 53% of FLT3-ITD-positive patients treated with quizartinib (75). Preliminary results of a phase II trial of patients ≥18 years old with relapsed or refractory AML showed an encouraging composite CR rates (CR + CRp + CRi) of 44%, with nearly one-third of patients successfully bridged to stem cell transplant (60). Phase I/II studies combining quizartinib with azacitidine or low-dose cytarabine are ongoing (61). Quizartinib is an oral agent and has been generally well tolerated, making it an exciting prospect for less fit patients with AML. Another multi-targeted tyrosine kinase inhibitor, ASP2215, is also in ongoing phase I trials (62).

Volasertib, a small molecule inhibitor of the polo-like kinase 1 (PLK1) protein, was combined with low-dose cytarabine and compared to low-dose cytarabine alone in a randomized, phase II trial for unfit patients with AML. The combination arm showed improved CR + CRi (31 versus 13.3%, *p* = 0.052) (57). The phase III trial is ongoing (58).

GSK2141795, a novel agent that blocks the phosphoinositide 3-kinase (PI3K)/AKT signaling pathway, is being studied in a phase II trial of unfit patients with RAS-mutated AML in combination with the MEK inhibitor trametinib (59).

Studies with Pevonedistat, a first-in-class inhibitor of NEDD-8 activating enzymes (NAE) in combination with azacitidine are ongoing. In phase I, this combination was safe and generally well tolerated. Among the 18 patients evaluable for response, a 56% ORR was reported (63). Phase II is underway.

### Epigenetic Therapies

Besides the DNA hypomethylating agents discussed above, other epigenetic therapies such as histone deacetylase inhibitors are also of interest in this population, and combination approaches are being evaluated (76). Phase II studies combining pracinostat with azacitidine in older adults with AML are accruing (65), and preliminary results are encouraging (77). The role of valproic acid, perhaps in combination with all-trans retinoic acid and other epigenetic modifiers, has yet to be fully elucidated (66). Other combinations of epigenetic therapy with different modalities continue to be studied. Examples include the topoisomerase-II inhibitor vosaroxin plus decitabine (68, 78) and bortezomib plus decitabine (79). Mutations in IDH1 and IDH2, which inhibit TET2 enzymatic function and thereby result in DNA hypermethylation, also represent novel epigenetic targets. Phase I studies of oral IDH1 and 2 inhibitors in patients with the respective mutations are ongoing (69, 70). DOT1L, a histone methyltransferase that plays a critical role in leukemic transformation induced by *MLL* rearrangements, is also a promising therapeutic target that is being investigated in early phase trials in *MLL*-rearranged leukemias (71, 80). Novel analogs and/or novel formulations of existing DNA methyltransferase inhibitors are also under active investigation in AML (72).

### Other Novel Agents

Selinexor, an oral selective inhibitor of nuclear export (SINE), is currently being investigated in a phase II randomized trial for unfit patients as a single agent versus physician’s choice, which includes hypomethylating agent or supportive care (73). There are also ongoing early phase trials investigating this agent in combination with chemotherapy or hypomethylating agent therapy.

Of course, the importance of supportive measures for patients undergoing less intensive therapy or no therapy should not be overlooked. Prophylactic antimicrobials, transfusion of blood products as needed, and hydroxyurea if needed for cytoreduction can all be utilized in an effort to reduce hospitalization rates in older patients with AML with the hope of improving quality of life.

## Conclusion

As the world’s population continues to age, the number of people diagnosed with AML each year can be expected to rise, adding urgency to the need for more effective and less toxic therapies for older, less fit adults. Available therapies outside of the realm of clinical trials are few. Clinical trial participation should be considered the standard of care for unfit patients and patients with high-risk disease whenever possible. Developing and validating uniform definitions for risk stratification according to fitness and integrating this within the context of disease biology are of utmost importance with regard to the design, implementation, and interpretation of clinical trial data in this patient population.

The criteria that define patients unfit for intensive induction chemotherapy are currently evolving and require validation. Therefore, at the present time, we recommend that clinicians incorporate the currently available tools described herein plus patient preferences in the development of treatment strategies for the individual patient.

## Author Notes

KP is a fellow in the section of hematology and oncology as well as clinical pharmacology at the University of Chicago. OO is an Associate Professor of Medicine in the section of Hematology and Oncology at the University of Chicago.

## Author Contributions

KP and TO both performed the literature review and authored the manuscript. The authors meet criteria for authorship as recommended by ICMJE. The authors received no direct compensation related to the development of the manuscript. All authors have read and approved the final manuscript.

## Conflict of Interest Statement

Olatoyosi Odenike has served on the advisory boards of Sunesis Pharmaceuticals, Algeta Pharmaceuticals, Spectrum Pharmaceuticals, Sanofi-Aventis, Incyte Pharmaceuticals, CTI/BioPharma, and Baxalta. Kristen Pettit has no conflict of interest to declare.

## Abbreviations

AML, acute myeloid leukemia; CCI, Charlson comorbidity index; CN-AML, cytogenetically normal acute myeloid leukemia; CR, complete remission; CRi, CR with incomplete hematologic recovery; CRp, CR with incomplete platelet recovery; ECOG PS, eastern cooperative oncology group performance status; ELN, European LeukemiaNet; EMA, European Medicines Agency; FDA, United States Food and Drug Administration; FLT3, FMS-like tyrosine kinase 3; GO, gemtuzumab ozogamicin; HCT-CI, hematopoietic cell transplantation-specific comorbidity index; ITD, internal tandem duplication; KPS, Karnofsky performance status; LDH, lactate dehydrogenase; MDS, myelodysplastic syndrome; NPM1, nucleophosmin 1; PI3K, phosphoinositide 3-kinase; PLK1, polo-like kinase inhibitor 1; PR, partial response; SPPB, short physical performance battery; WHO, World Health Organization.
